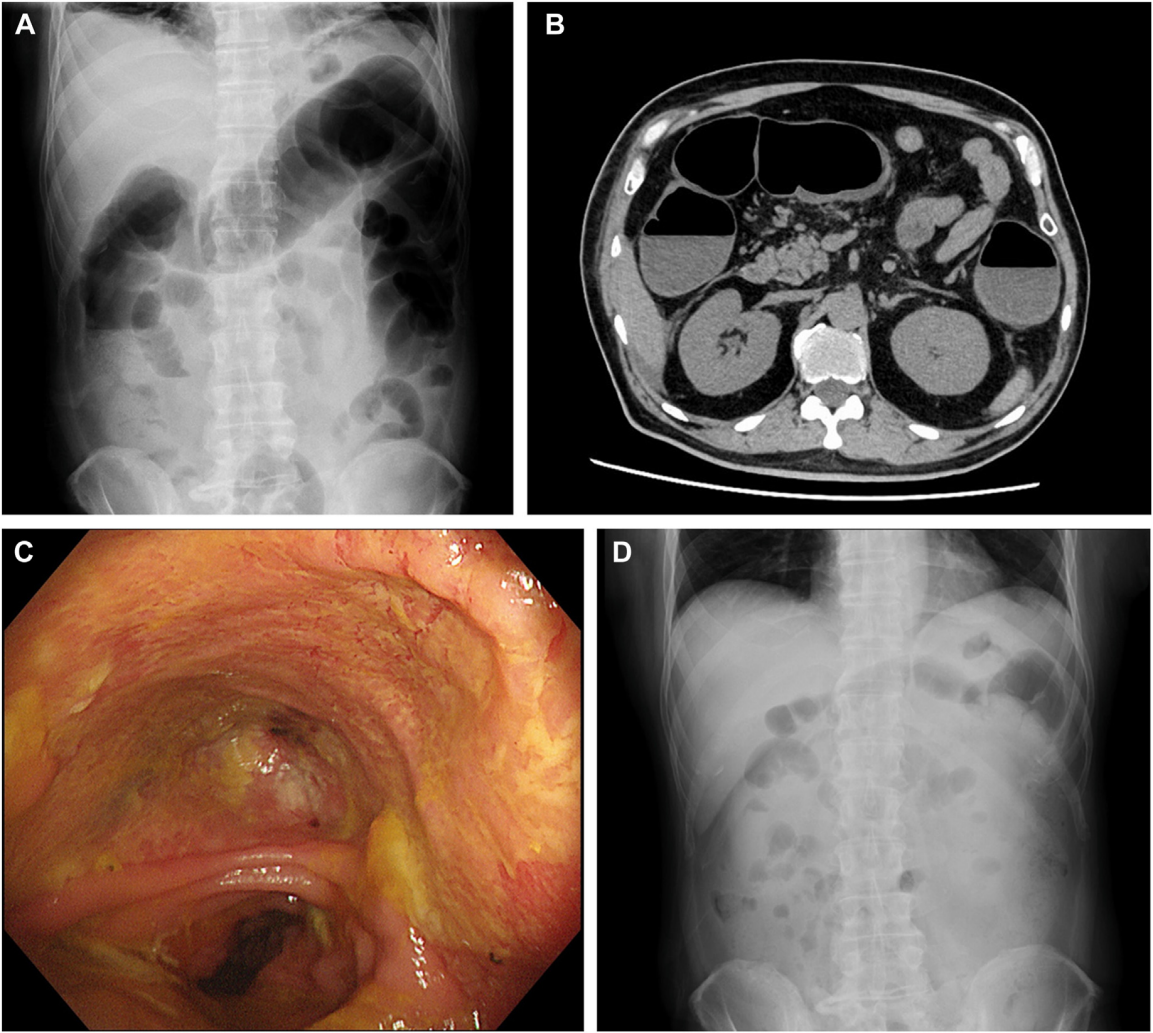# A Case of Successful Treatment Against Toxic Megacolon Associated With Immune Checkpoint Inhibitor-Induced Colitis

**DOI:** 10.1016/j.gastha.2023.10.009

**Published:** 2023-10-30

**Authors:** Sayaka Ikeda, Daisuke Watanabe, Yuzo Kodama

**Affiliations:** Division of Gastroenterology, Department of Internal Medicine, Kobe University Graduate School of Medicine, Kobe, Japan

A 53-year-old man was admitted due to severe diarrhea, subsequent to the second month of initiating a combination therapy of nivolumab and ipilimumab for malignant melanoma. Upon admission, he had a high fever of 38.0°C and elevated C-reactive protein. The computed tomography of his abdomen showed marked colonic dilatation (exceeding 7 cm), consistent with the diagnosis of toxic megacolon (Figure A and B). Colonoscopy showed continuous inflammation from the rectum throughout the colon, accompanied by deep-seated ulcers. Grade 3 colitis associated with immune-related adverse events was diagnosed, and steroid therapy (2 mg/kg/dy) was commenced. However, he experienced worsening of abdominal symptoms and a re-elevation of C-reactive protein, indicating unresponsiveness to the corticosteroid. Therefore, we began infliximab (IFX) therapy (5 mg/kg) on the 9th day poststeroid administration. A colonoscopy on the 13th day after the first IFX administration revealed epithelialization but with the presence of scattered deep ulcers (Figure C). A second IFX infusion was then performed on the 14th day after the initial IFX administration. Forty-two days after the initial IFX therapy, he was discharged as abdominal symptoms, signs of inflammation on blood tests, and the finding of toxic megacolon (Figure D) had being resolved.

**Figure:**